# Pharyngitis

**Published:** 1854-04

**Authors:** 


*Pharyngitis.*—Take Iodine ʒss., Iodide Potash ʒj., distilled water ℥j. Sugar and Gum Accacia *aa* ʒij. M. Dr. Merrill in the Boston Med. & Surg. Journal, recommends its application with a camel's hair pencil when a tickling sensation induces a desire to cough.

